# Non-linear effects and lag patterns of eco-environmental factors on scrub typhus: a spatiotemporal analysis in Yunnan, China, 2015–2022

**DOI:** 10.1186/s13071-026-07547-2

**Published:** 2026-07-09

**Authors:** Xiangnan Jing, Yuewei Ling, Ruonan Jing, Fuying Guo, Feifei Yin, Dongsheng Huang, Lijie Zhang, Xiangyu Yan, Tiejun Shui

**Affiliations:** 1 State Key Laboratory of Ecological Safety and Sustainable Development in Arid Lands, Northwest Institute of Eco-Environment and Resources, Chinese Academy of Sciences, Lanzhou, 730000 Gansu China; 2 University of Chinese Academy of Sciences, Beijing, 100049 China; 3 Department of Management Science and Engineering, Stanford University, Stanford, CA 94305 USA; 4 College of Forestry, Northwest A & F University, Yangling, Xianyang, 712100 Shaanxi China; 5 The People’s Hospital of Lincang, Lincang, 677000 Yunnan China; 6 School of Basic Medical Sciences, Key Laboratory of Tropical Translational Medicine of Ministry of Education, Hainan Medical University, Haikou, 571199 Hainan China; 7 Hainan Medical University-The University of Hong Kong Joint Laboratory of Tropical Infectious Diseases, Hainan Academy of Medical Sciences, Haikou, 571199 Hainan China; 8 Baoshan Center for Disease Control and Prevention, Baoshan, 678000 Yunnan China; 9 Chinese Center for Disease Control and Prevention, Chinese Center for Disease Control and Prevention & Chinese Academy of Preventive Medicine, Beijing, 102206 China; 10 School of Disaster and Emergency Medicine, Tianjin University, Tianjin, 300072 China; 11 Yunnan Center for Disease Control and Prevention, Kunming, 650022 Yunnan China

**Keywords:** Scrub typhus, Yunnan Province, Normalized difference water index, Poisson–DLNM, Wavelet analysis, Environmental drivers

## Abstract

**Background:**

Yunnan Province is characterized by a high incidence of scrub typhus and complex ecological environments, where epidemic patterns are shaped by the dual influence of the plateau monsoon system and geomorphological evolution. However, macro-scale meteorological indicators often fail to accurately capture climatic features within such rugged terrains, leaving the multi-factor coupled spatiotemporal associations of scrub typhus epidemics elusive.

**Methods:**

This study integrates Wavelet periodicity analysis and STL decomposition to identify the temporal rhythms and structural mutations of scrub typhus. Furthermore, GLM and Poisson–DLNM were employed to evaluate the independent contributions and non-linear lag risks of environmental factors.

**Results:**

Our findings indicate that scrub typhus in Yunnan Province exhibits steady-state seasonality characterized by a dominant 12-month cycle. The epidemic dynamics underwent a structural mutation around 2018, marking a transition into a high-intensity epidemic stage. In terms of environmental drivers, the Normalized Difference Water Index (NDWI) outperformed traditional precipitation indicators in risk explanatory power (aRR=1.93, 95% CI 1.84–2.04), suggesting that moisture accessibility in local micro-habitats is a critical variable for chigger survival. Furthermore, due to the saturation effect of the plateau biomass system, the Enhanced Vegetation Index (EVI) proved superior to the Normalized Difference Vegetation Index (NDVI) in characterizing habitats, with EVI showing a significant association (aRR = 0.75, 95% CI 0.72–0.78), while NDVI remained non-significant. Regarding human interference, habitat remodeling induced by urbanization has significantly intensified the human–rodent contact interface (aRR = 1.15, 95% CI 1.13–1.16). Finally, soil moisture exhibited the strongest cumulative risk effect (Cumulative RR = 2.23, 95% CI 1.86–2.67) characterized by a persistent long-term lag, and maintained a significant independent driving effect (aRR = 1.05, 95% CI 1.02–1.08).

**Conclusions:**

This study elucidates that in regions with complex topography, the core drivers of scrub typhus epidemics are constituted by local geohydrological indicators and micro-environmental evolution instead of macro-meteorological factors. The findings underscore the importance of integrating high-resolution geodetic monitoring indicators into infectious disease early-warning systems. These results provide a robust scientific foundation for the identification of cross-border health security risks and the development of precision early-warning strategies.

**Graphical Abstract:**

**Figure:**
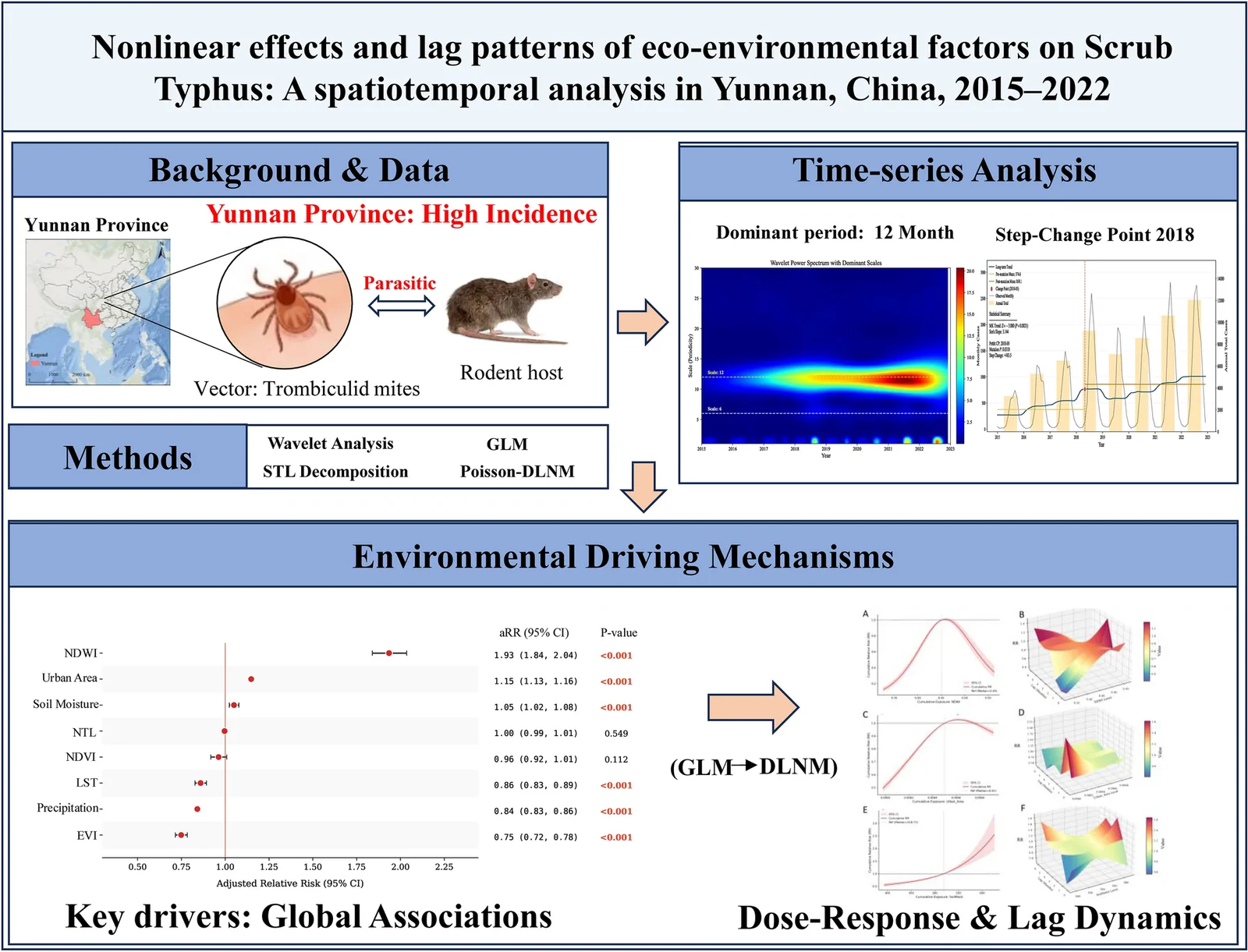

**Supplementary Information:**

The online version contains supplementary material available at https://doi.org/10.1186/s13071-026-07547-2.

## Background

Scrub typhus is a severe natural-focal infectious disease caused by *Orientia tsutsugamushi* and transmitted by the bite of larval trombiculid mites [1]. Historically confined to the “tsutsugamushi triangle” (Pakistan, Japan, and Australia), the disease now shows a clear global expansion trend linked to rising temperatures. Cases have recently emerged in South America, Africa, and parts of Europe [2, 3]. In China, the epidemic range has expanded significantly over the past two decades. Incidence rates continue to rise, with the epidemic center shifting from traditional southern regions toward northern and southwestern inland areas [4, 5]. Complex clinical presentations and high misdiagnosis rates, combined with the lack of an effective vaccine, make scrub typhus a critical public health challenge in the Asia–Pacific and globally [6].

The transmission dynamics of scrub typhus depend on a complex ecosystem comprising the pathogen, vector, host, and environment [7, 8]. As the sole vector, the chigger life cycle is highly sensitive to microclimate and habitat changes. In particular, soil moisture and land surface temperature govern egg hatching and larval activity [4, 9]. Meteorological factors regulate vector breeding rates and pathogen replication efficiency, while land-use changes, vegetation types, and landscape fragmentation reshape the spatial distribution of the disease by altering rodent host habitats [10, 11]. Under global warming and intense human intervention, the original ecological balance has been disrupted, leading to profound shifts in the spatial boundaries and seasonal patterns of natural scrub typhus foci [12–14].

While existing research links environmental factors to scrub typhus incidence [15, 16], these driving mechanisms often exhibit significant non-linearity, time-lag effects, and spatial non-stationarity across highly heterogeneous geographic units [17, 18]. Especially in regions with complex topography and diverse climates, the limitations of macro-meteorological indicators in characterizing local micro-habitats leave epidemic triggering mechanisms unclear [11]. Current research on infectious diseases and environmental change focuses heavily on conventional meteorological factors, such as precipitation and temperature, while the potential of high-resolution remote sensing indicators to capture near-surface micro-habitat water accessibility remains under-explored [19]. Furthermore, the lack of systematic assessments regarding the stability of scrub typhus epidemic rhythms and their structural mutations limits the accuracy of identifying outbreak risks near ecological tipping points, failing to meet the requirements for precision public health intervention [20].

To address these challenges, this study focuses on Yunnan Province, a region with high scrub typhus incidence and complex ecological environments. We integrated epidemiological surveillance data from 2015 to 2022 with multi-source high-resolution remote sensing variables. Wavelet periodicity analysis and Seasonal-Trend decomposition using Loess (STL) were employed to resolve the temporal rhythms, time–frequency evolution, and structural components of scrub typhus in Yunnan. In addition, Generalized Linear Models (GLM) and Distributed Lag Non-linear Models (DLNM) were combined to quantitatively evaluate the independent driving effects and non-linear lag risks of environmental factors. By comparing the risk assessment capabilities of various remote sensing indicators, this study aims to reveal how local geospatial and hydrological indices, rather than macro-meteorological factors, constitute core elements for maintaining natural foci. These findings provide a scientific basis for developing early-warning systems based on micro-environmental monitoring in Yunnan Province.

## Methods

### Study area

**Fig. 1:**
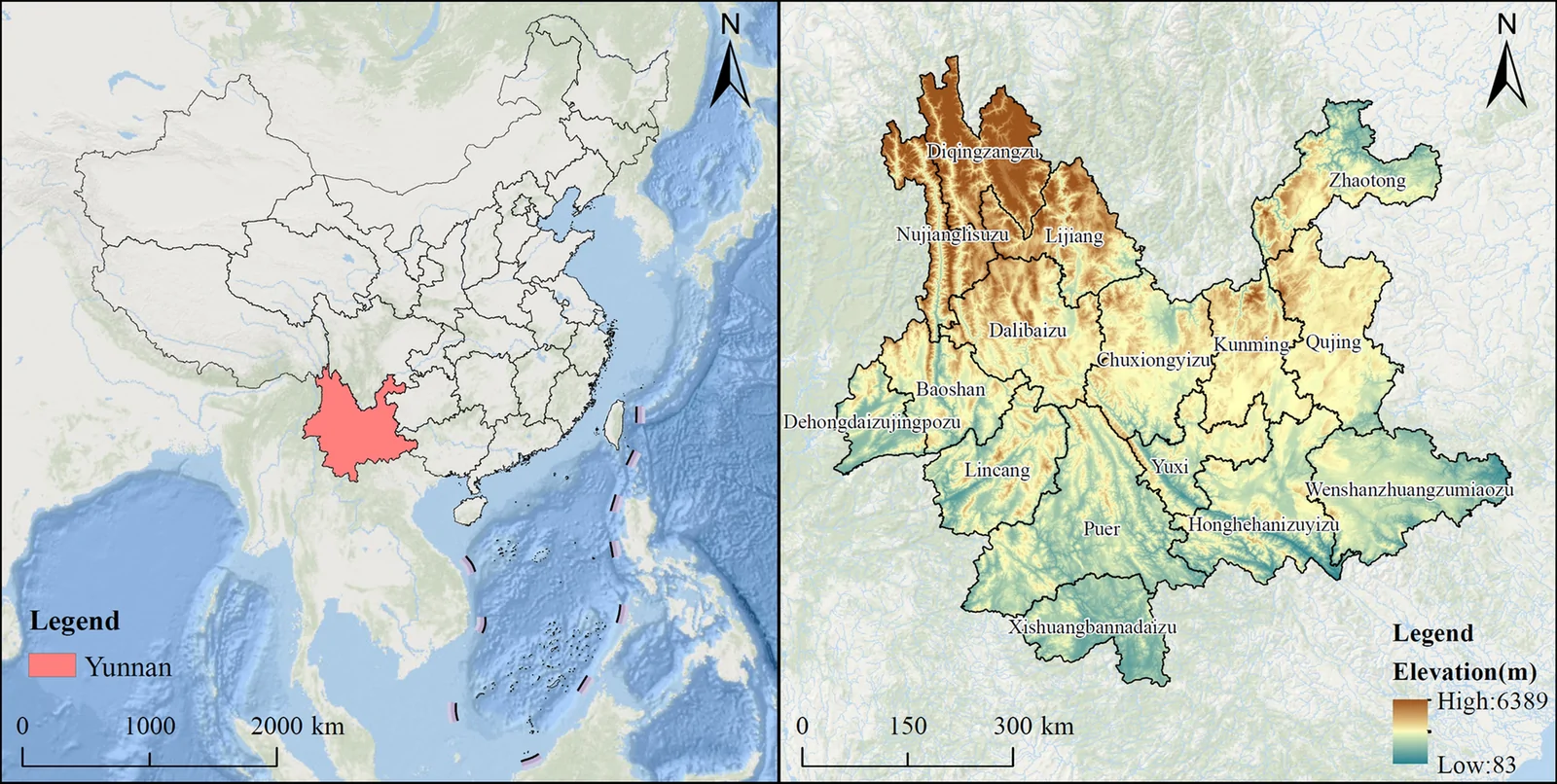
Geographical environment and topographic profile of Yunnan Province

Situated on the southwestern border of China, Yunnan Province exhibits a stepped topographical decline from northwest to southeast, creating unique vertical climatic zones and complex ecosystems (Fig. 1) [21]. The region is dominated by subtropical and tropical monsoon climates, characterized by abundant but unevenly distributed rainfall driven by the dual influence of the Indian and Pacific Oceans. Such intricate ecological structures provide not only ideal habitats for rodent hosts but also essential microclimatic barriers for the growth and proliferation of chigger mites [22]. Driven by monsoonal dynamics, the spatial distribution of scrub typhus in this area displays high heterogeneity. Consequently, investigating the spatial associations between geographic factors and scrub typhus epidemics in Yunnan is of significant scientific and public health value for developing precision early-warning models and regionalized control strategies.

### Data sources and ethics statement

Epidemiological data for scrub typhus cases in Yunnan Province from 2015 to 2022 were obtained from the surveillance system of the Chinese Center for Disease Control and Prevention. The study protocol was formally reviewed and approved by the Ethics Committee of the Yunnan Center for Disease Control and Prevention (Approval No. 2023-19). This research utilized only de-identified and county-level aggregate statistical data. All procedures were conducted in accordance with the ethical standards of the institutional and national research committee, and since the dataset contains no identifiable personal information, the requirement for informed consent was waived.

### Case definition and quality control

All scrub typhus cases included in this study were diagnosed in strict accordance with the national diagnostic criteria (WS 215-2008) issued by the National Health Commission of China. To ensure the accuracy and reliability of the findings, a rigorous data cleaning process was implemented. Only clinically diagnosed and laboratory-confirmed cases were included in the analysis. Suspected cases from the registry were excluded to minimize potential statistical bias resulting from diagnostic uncertainty.

### Meteorological and landscape indicators

As a typical natural-focal infectious disease, the transmission dynamics of scrub typhus are profoundly regulated by the complex topographical features, multidimensional climatic factors, and human activities in Yunnan Province. Regarding climatic drivers, this study utilized the GEE platform to extract monthly mean land surface temperature (LST) from the MODIS (MOD11A1, V061) product by capturing synchronous day-and-night thermal bands, applying a scale factor of 0.02 and unit conversions to quantify the regulatory effects of thermal conditions on chigger development, pathogen replication, and host activity [23, 24]. Hydrological conditions were comprehensively assessed using NASA GPM IMERG V07 precipitation data and GLDAS V021 Noah root-zone soil moisture to analyze the support capacity of the moisture environment for larval survival and surface micro-habitat ecological niches [25]. In terms of landscape and biomass dimensions, the MOD13Q1 (V061) product was employed to obtain Normalized Difference Vegetation Index (NDVI) and Enhanced Vegetation Index (EVI) arrays, while the Normalized Difference Water Index (NDWI) was calculated based on near-infrared and shortwave-infrared bands to accurately characterize vegetation dynamics and moisture barriers within host and vector habitats [26, 27]. To quantify the reshaping of natural foci by human activities, the proportion of urban area from MCD12Q1 (V061) extracted from the standardized IGBP classification layer (LC_Type1) and monthly VIIRS DNB (V2.1) nighttime light (NTL) data were used as socioeconomic proxy variables [28]. All environmental variables were extracted via the GEE platform and integrated with land-use and population distribution characteristics to evaluate biological exposure risks amidst environmental evolution, with detailed data sources provided in Table 1.

**Table 1:** . Multi-source environmental variables and data sources for scrub typhus modeling

Environmental variable	Sensor	Version	Spatial resolution
Land surface temperature (LST)	Terra MODIS (MOD11A1)	V 061	1 km
Precipitation	NASA GPM (IMERG)	V 07	11 km
Soil moisture	GLDAS Noah	V 021	0.25°
Normalized difference vegetation index (NDVI)	MOD13Q1	V 061	250 m
Enhanced vegetation index (EVI)	MOD13Q1	V 061	250 m
NDWI	MODIS	V 061	250 m/500 m
Urban area	MCD12Q1	V 061	500 m
Nighttime light (NTL)	VIIRS DNB	V 2.1	500 m

### Wavelet periodicity analysis

To address the non-stationarity and multi-scale evolutionary characteristics of scrub typhus infection sequences in Yunnan Province, this study employed Continuous Wavelet Transform (CWT) for wavelet period analysis to resolve the dynamic evolution of epidemic cycles [29]. The CWT offers multi-resolution properties in both time and frequency domains: it captures transient features of case outbreaks by automatically shrinking the window at high frequencies and identifies stable long-period fluctuations by stretching the window at low frequencies. The complex Morlet wavelet was selected as the mother wavelet to ensure an optimal balance between time and frequency localization, with the calculation formula defined as follows:

1 $${\psi}_{0\left(\eta \right)}={\pi }^{\left\{-\frac{1}{4}\right\}{e}^{\left\{i{\omega}_{0}\eta \right\}{e}^{\left\{-\frac{{\eta }^{2}}{2}\right\}}}}$$

where the dimensionless central frequency ${\omega}_{0}$ was set to 6 to achieve an optimal balance between time and frequency resolutions. This configuration ensures a stable linear mapping relationship between the wavelet scale α and the Fourier period T, thereby significantly enhancing the biological interpretability of the identified scrub typhus cycles.

To quantify the distribution patterns of scrub typhus epidemic energy along the timeline and its temporal evolution across different time scales, this study constructed the Wavelet Power Spectrum (WPS) based on the aforementioned continuous wavelet transform [30]. This power spectrum intuitively characterizes the dual evolution of signal energy over time and frequency, thereby identifying instantaneous fluctuations in epidemic intensity and the dominant cycles at various scales. Finally, a red noise background generated via first-order autoregression was established as the benchmark for null hypothesis testing. Through Monte Carlo simulations, significant epidemic cycles passing the 95% confidence level test were extracted to define the core scrub typhus frequencies and time-varying characteristics within Yunnan Province, with the calculation formula defined as follows:

2 $${P}_{W\left(a,b\right)}={\left|{W}_{f\left(a,b\right)}\right|}^{2}$$

where the dimensionless central frequency ${\omega}_{0}$ was set to 6 to achieve an optimal balance between time and frequency resolutions. This configuration ensures a stable linear mapping relationship between the wavelet scale α and the Fourier period T, thereby significantly enhancing the biological interpretability of the identified epidemic cycles. Where ${P}_{W\left(a,b\right)}$ is the wavelet power spectrum value and ${W}_{f\left(a,b\right)}$ represents the wavelet transform coefficient. Here, *a* is the scale factor corresponding to frequency-domain characteristics, used to reflect the seasonal and multiannual epidemic cycles of scrub typhus in Yunnan Province, and *b* is the translation factor corresponding to time-domain characteristics, representing the position of the wavelet window as it shifts along the time axis.

### Generalized linear model

To address the typical integer-count characteristics and non-normal distribution of scrub typhus cases in Yunnan Province, this study constructed Generalized Linear Models (GLM) to quantitatively evaluate the driving effects of various environmental factors on the epidemic[31]. The GLM framework comprises a random component, a linear predictor, and a link function, with the formula defined as follows:

3 $$g\left(\mu \right)={\beta}_{0}+{\sum}_{i=1}^{n}{\beta}_{i}{X}_{i}$$

where μ is the expected number of scrub typhus infections in Yunnan Province, $g\left(\bullet \right)$ denotes the log link function, and ${X}_{i}$ represents the environmental factors utilized in this study. Considering the overdispersion observed in the scrub typhus data, this study employed the Poisson distribution as the random component and confirmed the applicability of the Poisson model at the current data scale by calculating the dispersion parameter[32].

In factor identification, *P* < 0.05 was established as the criterion for statistical significance. The *Adjusted Relative Risk* (*aRR*) was derived by calculating the exponential transformation of the regression coefficients. An *aRR* > 1 indicates that the factor serves as a risk factor promoting the occurrence of scrub typhus, while an *aRR* < 1 identifies it as a protective factor. Finally, the goodness-of-fit was evaluated using the Akaike Information Criterion (AIC) to select the primary drivers that contribute significantly to the scrub typhus epidemic in Yunnan Province[33].

### Distributed lag non-linear model

Considering that the impact of environmental factors on scrub typhus exhibits prominent non-linear characteristics and significant temporal lags due to the environment–vector–host ecological cycle, this study integrated the Distributed Lag Non-linear Model (DLNM) based on the aforementioned GLM [34]. The primary objective was to resolve the complex dynamic effects of environmental drivers across both exposure intensity and lag time dimensions, thereby comprehensively revealing the long-term driving mechanisms of environmental evolution on scrub typhus. The core innovation of DLNM lies in the construction of a cross-basis function. This approach combines the basis functions of the exposure–response and lag-response dimensions through a tensor product to capture non-linear risks and time-varying patterns within a three-dimensional space. Furthermore, the *Cumulative Relative Risk* (*Cumulative RR*) was calculated by integrating the effects across the entire lag period to quantify the total net impact of specific environmental stressors on the scrub typhus [35]. The calculation formula is defined as follows:

4 $$g({\mu}_{t})=\alpha +\omega ({x}_{t,l},\beta )+\sum {\eta}_{j}{C}_{jt}+{\epsilon}_{t}$$

where ${\mu}_{t}$ represents the expected number of scrub typhus cases in Yunnan Province at time t, $\omega ({x}_{t,l},\beta )$ denotes the cross-basis matrix of the core environmental factors, and ${C}_{jt}$ refers to the covariates utilized to control for long-term trends and seasonal fluctuations.

Eliminating reporting artifacts across regions, the total population of each spatial unit was explicitly incorporated into all regression models, ensuring that the estimated risk metrics are fully population-adjusted. To balance model flexibility and robustness, natural cubic splines were applied in both the exposure–response and lag-response dimensions of the DLNM. This specification allows the model to characterize potential non-linear associations and delayed effects of environmental factors on scrub typhus incidence. A maximum lag period of 6 months was specified based on the ecological characteristics of scrub typhus transmission and regional climatic conditions. Previous studies have suggested that meteorological influences on scrub typhus may operate through delayed ecological processes involving vegetation growth, rodent host dynamics, and chigger population development, resulting in lagged effects that can extend over several weeks to several months [36, 37]. In addition, a 6-month lag window encompasses the transition between the dry and wet seasons in Yunnan Province, allowing the model to assess both short-term and cumulative seasonal effects of environmental variability.

### Experimental setup

All data preprocessing and statistical modeling tasks in this study were executed on a high-performance workstation equipped with an Intel Core i9 processor. The construction of all statistical models and the integrated processing of GEE geospatial data were developed and implemented within a Python 3.9 environment. To ensure the accuracy and reproducibility of the research results, the experimental computational workflows were optimized to maintain high operational efficiency during parameter iteration across large-scale, multi-source datasets. The specific configurations and detailed structural parameters of these statistical models are systematically summarized in Table S1.

## Results

**Fig. 2:**
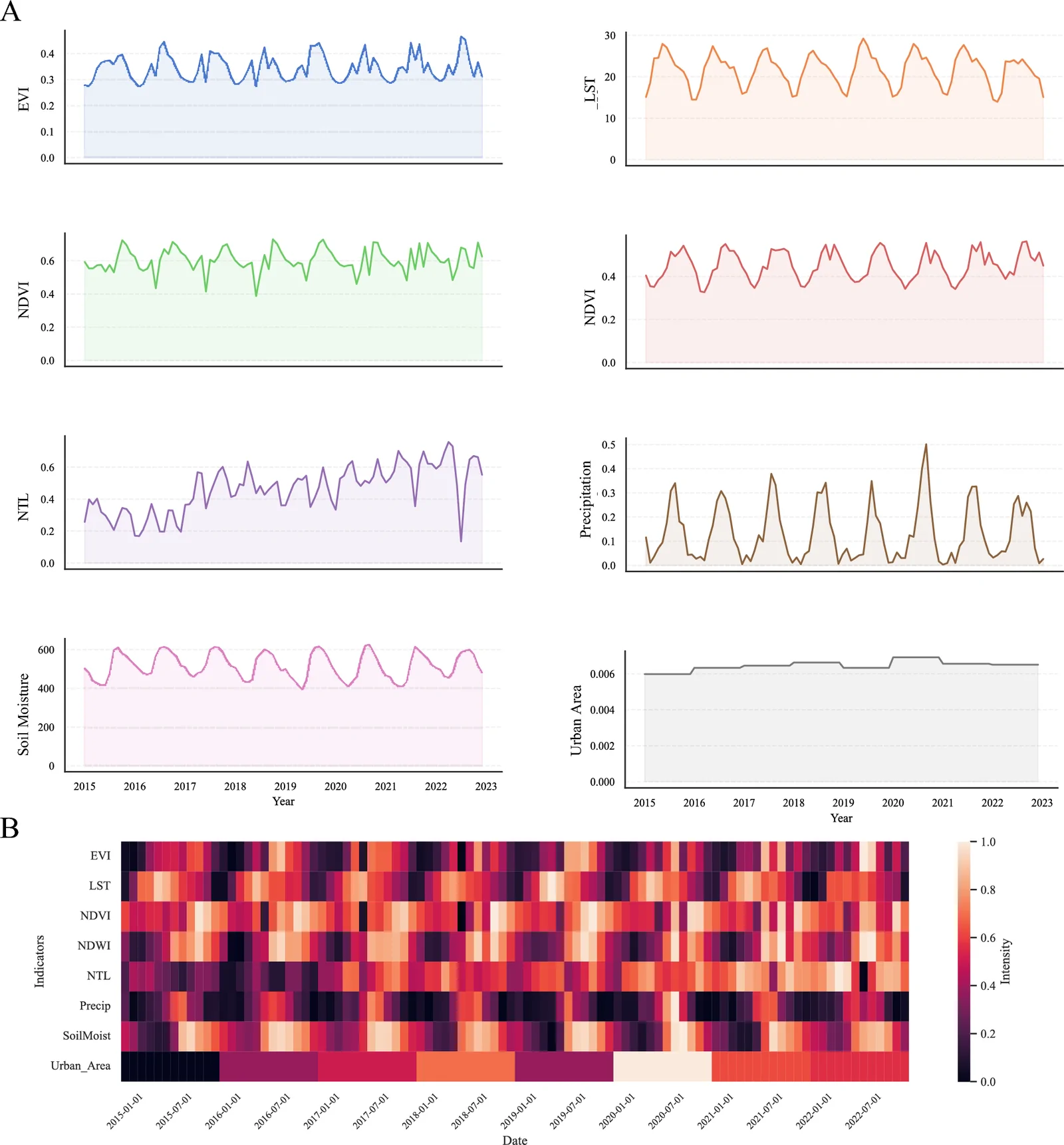
Spatiotemporal evolution of environmental and socioeconomic indicators in the study area from 2015 to 2022. **A** Time-series variation of the selected indicators; **B** heatmap of multi-indicator dynamics based on normalized intensity

Between 2015 and 2022, a total of 62,554 confirmed cases of scrub typhus were recorded in Yunnan Province. Figure 2 illustrates the temporal dynamics and correlations of the eight key indicators involved in this study. As shown in Fig. 2A, LST, Precipitation, NDVI, and EVI exhibit significant and regular seasonal fluctuations, revealing a stable natural phenological rhythm in the Yunnan region. Meanwhile, NTL and Urban Area demonstrate a distinct stepwise upward trend, characterizing the continuous expansion of urbanization. The heatmap in Fig. 2B further reveals that the six natural environmental factors exhibit highly consistent fluctuations in the vertical dimension, reflecting prominent synchronous hydrothermal characteristics and ecological cycling. The NTL and Urban Area indicators steadily transition from low intensity in the early stages to high intensity in the later periods, recording the irreversible growth of socioeconomic activities. This representation integrates periodic natural evolution with the linear expansion of human activities within a unified spatiotemporal dimension, clearly revealing the coupling relationship between environmental factors and socioeconomic indicators. This provides intuitive data support for an in-depth exploration of the mechanisms by which urban expansion influences the transmission of scrub typhus.

**Fig. 3:**
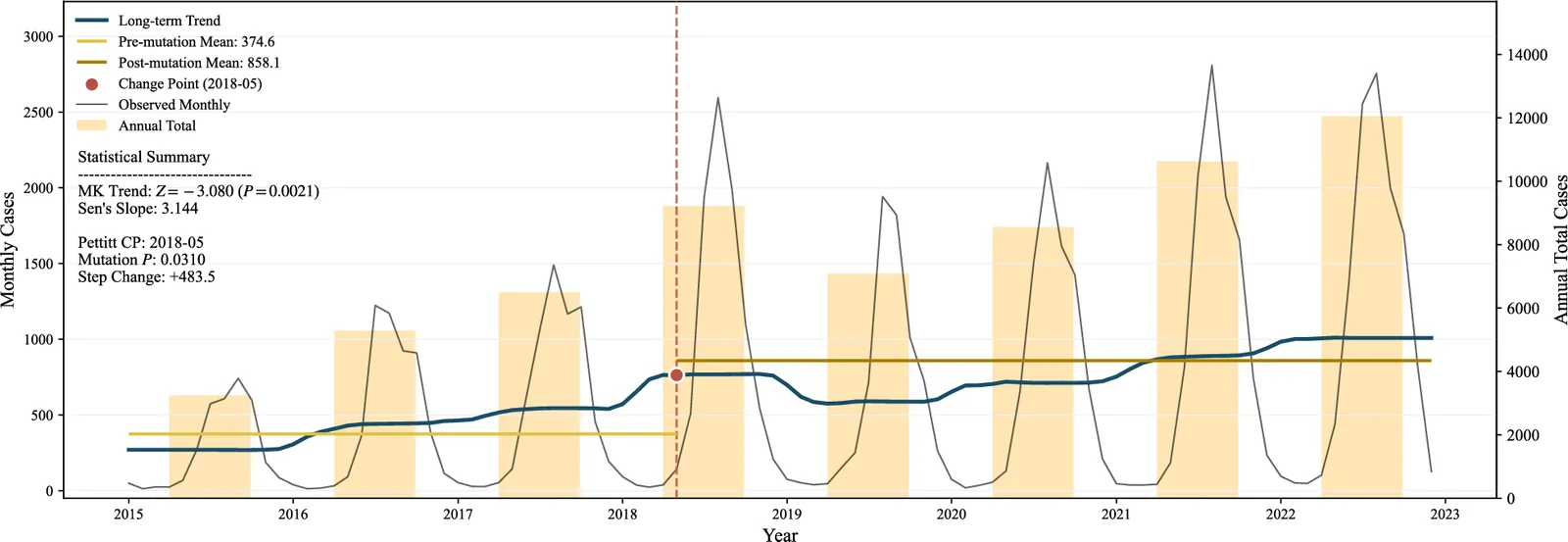
Trend analysis and mutation detection of reported scrub typhus cases in Yunnan Province, 2015–2022

Monthly reported scrub typhus cases in Yunnan Province were marked by pronounced seasonal fluctuations and a distinct phased growth characteristic across the 2015–2022 study interval. The STL decomposition results indicate that after removing seasonal components, the long-term trend follows a monotonic upward trajectory. The Mann–Kendall test confirms the statistical robustness of this rising trend (*Z* = 3.08, *P* = 0.002), while the Sen’s Slope of 3.144 verifies the sustained growth rate of scrub typhus during this period. Furthermore, the Pettitt test identified a significant structural mutation in the sequence occurring in May 2018 (*P* < 0.01), after which the incidence levels demonstrated a notable step-change. With 2018 serving as a demarcation point, the average monthly cases in Yunnan Province surged from 374.6 in the early stage to 858.1 in the later stage, representing a 129.1% increase. Trend analysis suggests that since May 2018, the epidemic intensity of scrub typhus in Yunnan Province has remained significantly higher than the pre-mutation baseline, entering a phase of sustained high incidence (Fig. 3).

**Fig. 4:**
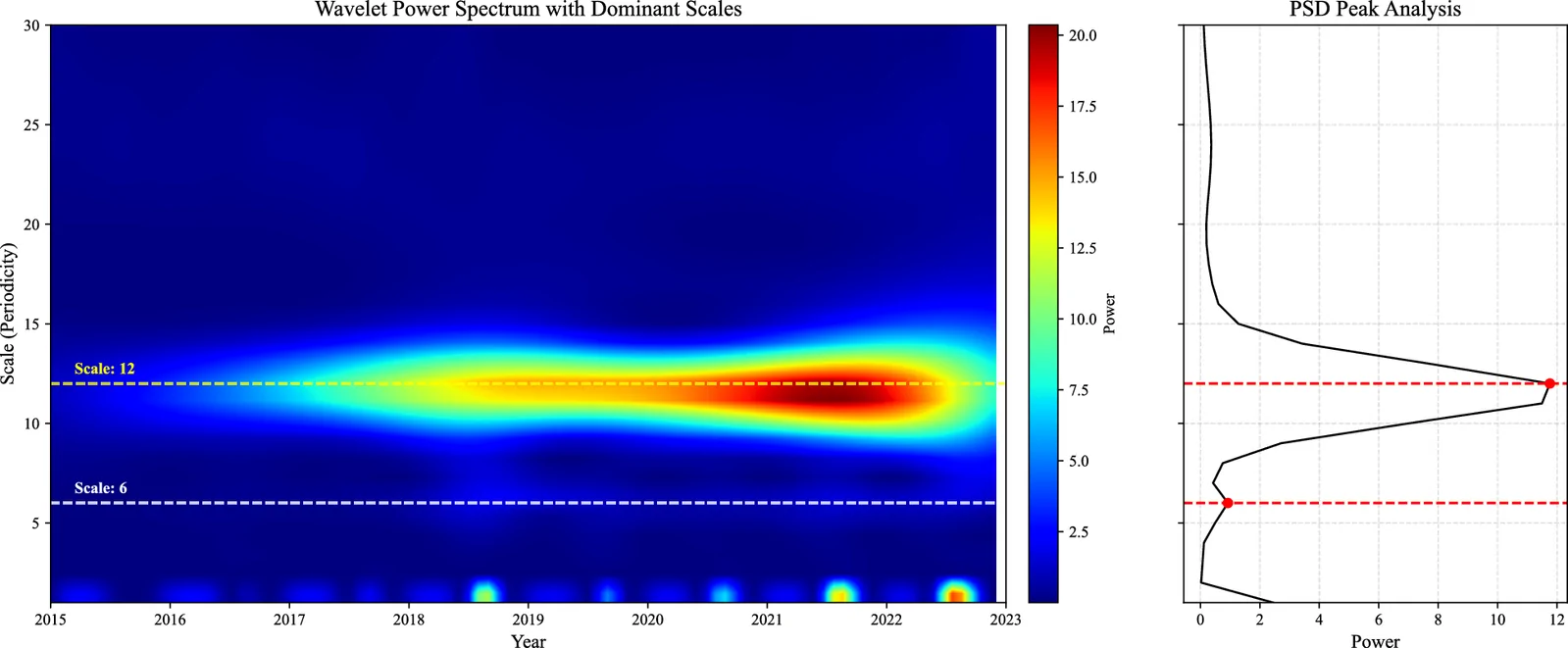
Wavelet analysis of scrub typhus incidence from 2015 to 2022. The left panel shows the continuous wavelet power spectrum, where the *x*-axis represents the year and the *y*-axis represents the scale (periodicity). The color gradient from blue to red indicates increasing power intensity (amplitude of incidence fluctuations). White and yellow dashed horizontal lines identify the dominant periodic scales (Scale 12 and Scale 6). The right panel illustrates the global wavelet power spectrum (PSD), representing the average energy distribution across all scales, with red dots and dashed lines highlighting the most significant periodic peaks

Figure 4 illustrates the continuous wavelet transform and global power spectrum analysis, reveals that the epidemic exhibits a significant annual seasonality, dominated by a 12-month primary cycle alongside a weaker 6-month secondary periodicity. The time–frequency evolution of the wavelet power spectrum indicates that the intensity of periodic signals fluctuated markedly throughout the study period. Specifically, from 2015 to 2018, the annual cycle energy remained relatively weak, reflecting a comparatively stable fluctuation of scrub typhus in Yunnan. However, starting in 2019, the energy intensity at Scale 12 significantly strengthened and the frequency band widened, suggesting a surge in seasonal epidemic intensity and a pronounced escalation in risk during this phase. This time–frequency characterization precisely delineates the stability of the incidence rhythm and the pivotal turning points in epidemic intensity, providing a scientific basis for public health authorities to forecast disease trends and optimize the allocation of prevention and control resources.

**Fig. 5:**
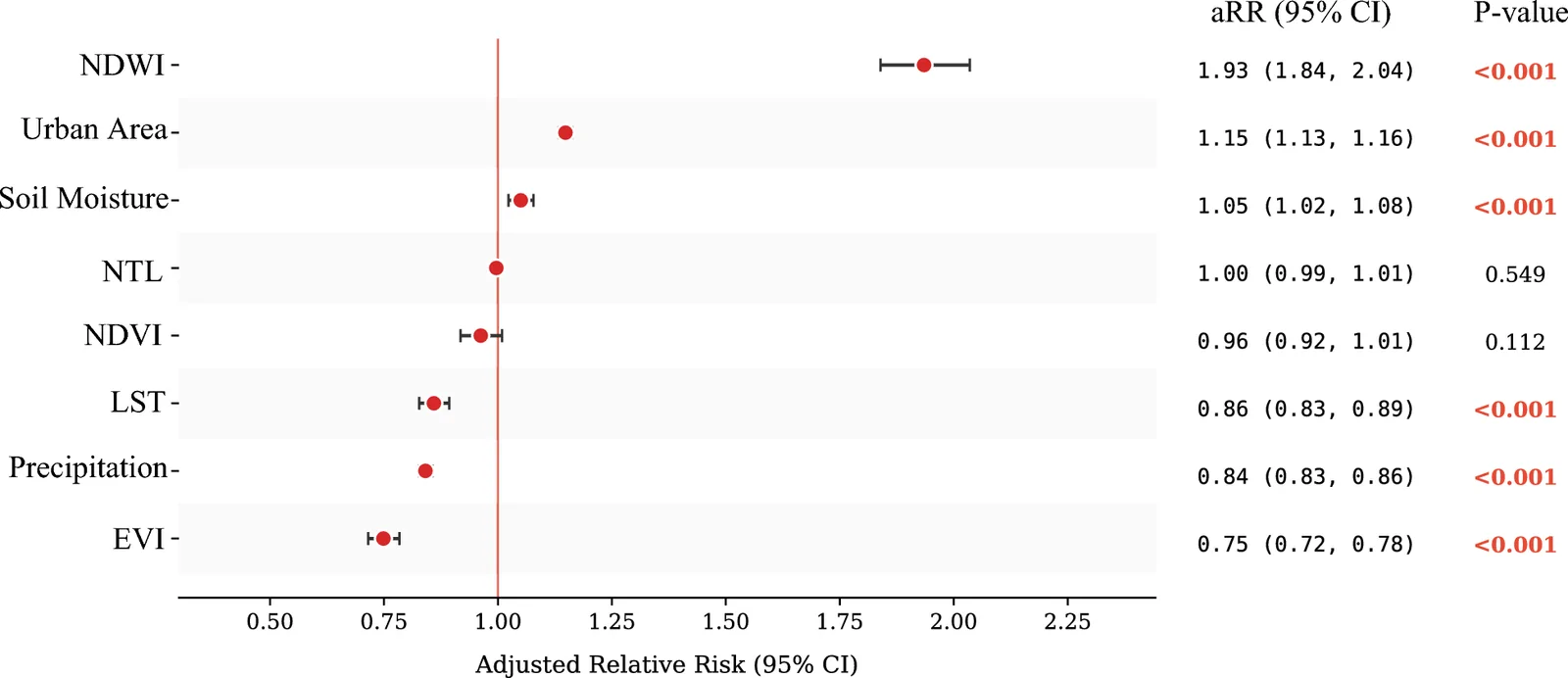
Associations between environmental factors and scrub typhus risk based on the GLM. Results are presented as *aRRs* with 95% confidence intervals (CIs). The red dots signify the *aRR* estimates, and the horizontal error bars represent the 95% CIs. The vertical red line indicates the null effect (*RR*=1.0). Factors with *P*<0.05 are considered statistically significant

GLM regression reveals that the spatial distribution of scrub typhus in Yunnan Province is modulated by distinct geographic drivers (Fig. 5). NDWI emerged as the most potent positive predictor (*aRR* = 1.93, 95% CI 1.84–2.04), underscoring surface moisture as a fundamental determinant governing chigger survival and subsequent transmission. While Urban Area (*aRR* = 1.15, 95% CI 1.13–1.16) and Soil Moisture (*aRR* = 1.05, 95% CI 1.02–1.08) exhibited significant risk-enhancing effects, EVI, LST, and Precipitation displayed negative associations, suggesting their net impact tends to suppress incidence after accounting for shared environmental variance. The lack of statistical significance for NDVI and NTL (*P* > 0.05) implies that transmission risk in this region is more sensitive to micro-scale vegetation structures and specific land-use configurations than to generalized greenness or macroeconomic intensity. To verify that these geographic risk attributions were not artifactual products of overdispersion inflation, we executed a rigorous parallel cross-validation using a Negative Binomial (NB) model. The empirical diagnostics demonstrated exceptional architectural stability, as all core eco-environmental and socioeconomic drivers consistently maintained identical effect orientations and extreme statistical significance across both statistical channels (Table S2). Furthermore, a formal auxiliary regression test via the Lagrange Multiplier framework was implemented to explicitly quantify the potential overdispersion scale [38]. The testing yielded a highly constrained overdispersion coefficient of *d* = 0.0576 (*P* < 0.001), mathematically confirming that while overdispersion is statistically present, its structural magnitude is strictly confined to a micro-scale level and does not introduce systemic bias to our core relative risk estimations. This complete empirical symmetry fully supports the robustness of our data attributions and justifies the retention of the original standardized Poisson framework.

**Fig. 6:**
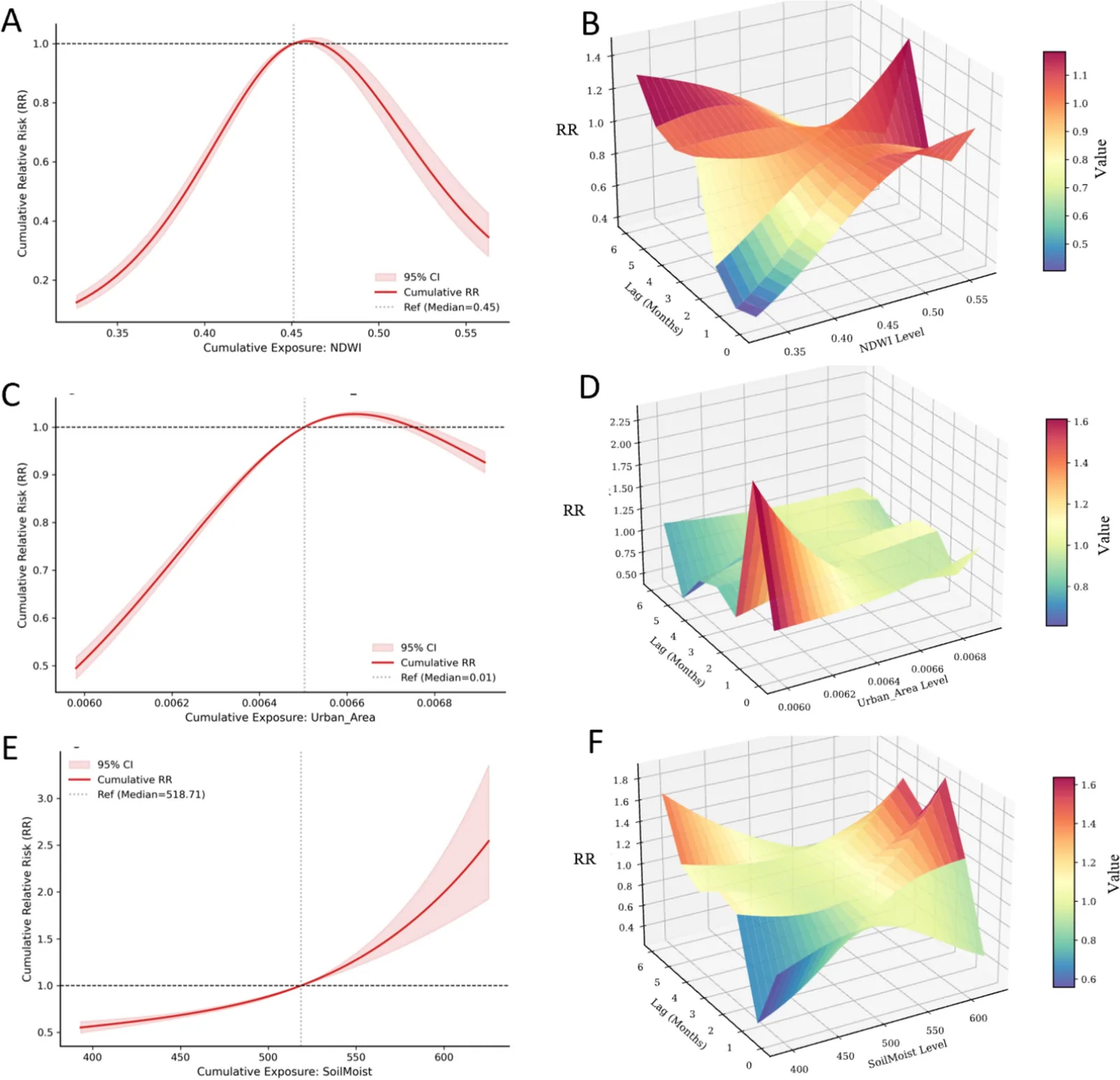
Distributed lag non-linear effect analysis of core environmental drivers identified by GLM on scrub typhus risk. The left panels **A**, **C**, **E** display the cumulative exposure–response curves, illustrating the overall cumulative impact of environmental factors on scrub typhus risk across the entire lag period (0–6 months). The solid red lines represent the estimated *Cumulative RR* and the vertical gray dashed lines indicate the reference values (medians of each factor). The right panels **B**, **D**, **F** present the corresponding 3D response surface plots, revealing the interactive driving characteristics of environmental exposure levels and lag times on the risk, with the color gradient from blue to red representing RR values ranging from low to high

To quantitatively resolve the non-linear and lagged driving mechanisms of environmental factors on scrub typhus, this study employed DLNM to conduct an in-depth analysis of the top three core drivers identified by the GLM. Cumulative exposure curves (Fig. 6A, C, E) reveal significant dose–response associations; with the median of each factor as a reference, the *Cumulative RR* for NDWI and Urban Area exhibited a distinct inverted U-shaped pattern, peaking near their respective median levels before declining at higher exposure levels. The risk associated with NDWI was significantly attenuated at extreme exposure (*Cumulative RR* = 0.38, 95% CI 0.33–0.44), while Urban Area displayed a slight protective effect (*Cumulative RR* = 0.95, 95% CI 0.93–0.98). In contrast, Soil Moisture exerted a potent risk-enhancing effect, with cumulative risk escalating alongside exposure intensity and reaching its maximum at the extreme level (*Cumulative RR* = 2.23, 95% CI 1.86–2.67). Figure 6B, D F further characterizes the interaction between exposure intensity and lag time, illustrating that the lagged association of NDWI is highly immediate, whereas Urban Area exhibits a short-term delay of approximately 1 month. Conversely, the transmission risk associated with Soil Moisture demonstrates strong persistence and long-lag characteristics; under high-exposure conditions, its single-month driving effect peaks at a 2-month lag, with the risk remaining significant throughout extended lags of 4–6 months. These findings confirm that the coupling of spatiotemporal lag effects and non-linear dynamics governs the evolution of scrub typhus risk, providing a critical scientific basis for identifying high-risk habitats.

## Discussion

Characterized by pronounced vertical zonation and a sharply defined wet–dry seasonality, Yunnan harbors a complex plateau monsoon system. This unique hydrothermal coupling serves as the fundamental ecological catalyst for scrub typhus epidemics, primarily by restructuring host habitats and modulating vector phenology. In this study, we sequentially implemented STL decomposition and wavelet analysis to resolve the temporal trajectories and multi-scale periodic signatures of the disease. These methods were further integrated with GLM and DLNM frameworks to quantitatively decouple the driving effects and spatiotemporal lag characteristics of specific environmental determinants.

The reconfiguration of epidemic dynamics constitutes a pivotal observation of this study. Wavelet analysis reveals a steady seasonal rhythmicity in scrub typhus incidence across Yunnan, characterized by a primary 12-month cycle and a secondary 6-month cycle. This underscores the fundamental regulatory role of low-latitude plateau phenology on the “host–vector–pathogen” chain. Such findings align closely with the phenological coupling mechanisms observed in tropical Southeast Asia, confirming the universality of monsoon-regulated infectious disease rhythms in low-latitude regions [39, 40]. Furthermore, STL decomposition integrated with the Pettitt test identified a structural breakpoint around 2018, marking a transition of epidemic dynamics from a stable phase into a multiplication stage. The 2018 step change may reflect a coupling of true epidemiological shifts and artifactual surveillance dynamics. While statutory reporting protocols remained uniform under the WS 215-2008 standard throughout 2015–2022, local diagnostic capacities and clinical awareness in Yunnan expanded around 2018. The gradual integration of molecular tools and continuous public health training likely helped reduce historical misclassification and under-reporting, contributing to the sharp increase in recorded cases. This breakpoint may concurrently reflect a genuine ecological shift, where cumulative hydroclimatic stressors crossed a critical biological threshold to drive the expansion of natural disease foci. This regime shift suggests that the non-linear accumulation of environmental drivers has reached an ecological tipping point [41], causing epidemic risk to exhibit non-linear acceleration rather than simple linear summation once critical thresholds are breached [42, 43].

Global patterns indicate that scrub typhus risk scales with rising temperature and precipitation, typically manifesting a 1–4-month lag tied to the *Leptotrombidium* mite life cycle [44, 45]. While remote sensing has confirmed a broad positive correlation between NDVI and mite distribution [46, 47], the driving mechanisms exhibit intense spatial heterogeneity in complex terrains like Yunnan due to divergent topographic and vegetative feedbacks. By stripping seasonal noise through a GLM, we identified NDWI as the primary driver, suggesting that surface moisture enrichment is more critical for mite survival than macro-precipitation in plateau landscapes; this finding refines the precipitation-centric models used in South China by capturing micro-habitat moisture accessibility [48, 49]. Crucially, the cumulative exposure architecture resolved by the DLNM revealed a distinctive inverted U-shaped non-linear risk trajectory for NDWI, peaking near its median level before showcasing significant risk attenuation under extreme saturation scenarios. This non-linear feedback loop can be robustly interpreted through the distinct micro-environmental requirements governing both vector and host ecology. Within the upward limb of the curve, incremental surface moisture enrichment enhances soil humidity and fuels local biomass production, which creates ideal microclimatic barriers for chigger egg hatching and larval quests while simultaneously expanding forage availability to support higher rodent population densities [50]. However, once the hydrological envelope crosses a critical biological threshold into extreme NDWI saturation, the environmental feedback reverses. Severe over-saturation or localized flooding directly disrupts the fragile micro-habitats of soil-dwelling trombiculid mites, drowning chigger nests and inducing substantial larval mortality [51]. Excessive pooling forces rodent hosts to abandon low-lying burrow networks and migrate away from hyper-saturated agricultural fringes, structurally collapsing the local human–rodent–vector contact interface and culminating in the observed protective effect at the upper exposure bound [52]. The risk associated with Urban Area echoes observations that urbanization reconfigures peri-urban fringes to facilitate spillover [11, 53], a trend consistent with the rural-to-urban diffusion seen in temperate regions [54]. The lack of statistical significance for NTL (*p* = 0.549), contrasted with the significant risk-enhancing effect of Urban Area (aRR = 1.15, 95% CI 1.13–1.16), implies that scrub typhus transmission in Yunnan is more sensitive to the physical reshaping of land-use configurations than to generalized macroeconomic intensity. Unlike temperate epidemics modulated by population aging [55], the patterns in Yunnan are defined by occupational exposure resulting from fragmented fringe habitats that overlap spatially with intensive agriculture. Unlike leisure-based exposure in parks, Yunnan’s workforce frequently encroaches upon mite habitats during irrigation and harvesting, increasing environmental dependency [56, 57]. Vegetation indices displayed significant heterogeneity in our model. Specifically, while EVI showed a negative correlation, traditional NDVI failed to reach statistical significance (*aRR* = 0.96, 95% CI 0.92–1.01). This divergence reflects the saturation effect inherent in high-biomass plateau systems. In densely canopied forests, NDVI loses sensitivity as values approach unity. In contrast, EVI is corrected with the blue band to effectively penetrate atmospheric interference and capture subtle structural changes in vegetation closely linked to mite habitats [58, 59]. From an eco-epidemiological perspective, the pronounced negative association of EVI implies that highly dense, undisturbed sylvatic forest canopies act as an ecological barrier rather than a risk catalyst. In these localized climax communities, the hyper-stable microclimatic buffering and high biodiversity support complex predator–prey networks that exert strong biological control over rodent reservoirs [60]. Fragmented fringe vegetation and highly disturbed, transitional shrublands, which are characterized by moderate EVI metrics, reshape the landscape into optimal ecotones that heavily facilitate human–rodent contact and vector proliferation, thereby magnifying spillover dynamics [61]. Finally, the DLNM results for Soil Moisture confirm a potent cumulative risk with long-lag signatures, quantifying micro-environmental drivers previously overlooked in the literature [62, 63].

This study offers clear practical implications for the precision prevention and control of scrub typhus in complex low-latitude plateau terrains. The robust cumulative effect exhibited by Soil Moisture serves as a more stable predictive source than traditional precipitation indices, supporting the development of high-precision early-warning systems. Given Yunnan’s strategic location adjacent to the Southeast Asian “Tsutsugamushi Triangle”, this research directly responds to the call for enhanced transboundary health security monitoring, providing an empirical basis for shifting epidemic prevention resources from universal coverage toward targeted deployment [54, 64]. However, several limitations should be noted. First, as a county-level ecological study, the observed associations represent population-level correlations rather than individual-level relationships, which may introduce the risk of ecological fallacy. Second, our findings could be influenced by unmeasured confounding, as granular variables such as individual behavioral patterns, precise agricultural exposure, and host control interventions were unavailable. Due to these constraints and the lack of long-term field monitoring data on host and vector pathogen-carrying rates, a complete closed-loop feedback mechanism at the micro-ecological level could not be reconstructed. To mitigate these issues, we utilized high-resolution remote sensing indicators (250m NDWI and EVI) and incorporated temporal splines in the DLNM to capture micro-habitat dynamics and control for time-varying confounders. Future research should integrate higher-resolution earth observations with field entomological surveillance and cross-border collaboration to better predict the climate-driven expansion of natural foci [8, 37].

## Conclusions

Our findings demonstrate that scrub typhus in Yunnan Province exhibits a significant 12-month seasonal rhythm, triggering an ecological tipping point in 2018 that marked a transition from a stable phase to a multiplication stage. In regions characterized by pronounced topographic heterogeneity, NDWI exerts a driving force on infection risk that far exceeds that of macro-precipitation, serving as a core determinant for the maintenance of natural foci in Yunnan. Furthermore, Soil Moisture, with its potent cumulative risk and long-lag signatures, demonstrates a robust capacity for capturing micro-environmental dynamics. This study highlights the necessity of employing EVI in high-biomass regions; its blue-band correction effectively overcomes the saturation effects inherent in traditional NDVI, allowing for a more precise characterization of subtle vegetation structural changes linked to vector habitats. In addition, the reconfiguration of fringe habitats induced by urbanization has significantly elevated infection risks among occupationally exposed populations. Collectively, these results elucidate the non-linear and lagged driving mechanisms of geographic factors, providing a scientific foundation for implementing targeted ecological interventions and transboundary health security monitoring in the context of global climate change.

## Supplementary Information

Supplementary Material 1.

## Data Availability

Data supporting the main conclusions of this study areincluded in the manuscript.
