# Supplementary material for: Non-linear effects and lag patterns of eco-environmental factors on scrub typhus: a spatiotemporal analysis in Yunnan, China, 2015–2022
Source: Parasit Vectors. 2026 Jul 9;19:404. doi: 10.1186/s13071-026-07547-2 (PMC13599320; doi:10.1186/s13071-026-07547-2)
Supplement: Supplementary file 1 [file 13071_2026_7547_MOESM1_ESM.docx]

| **Table S1**. Hyperparameter profiles and model specifications for the check framework | | | |
| --- | --- | --- | --- |
| Model | Model Parameter | Specification | Parameter Value |
| GLM | Number of Observations | Total consecutive monthly data points | 96 |
|  | Df Model | Degrees of freedom allocated for predictors and seasonal covariates | 11 |
|  | Df Residuals | Degrees of freedom remaining for robust error estimations | 84 |
|  | Estimation Method | Iteratively Reweighted Least Squares algorithm | IRLS |
| DLNM | Exposure-response Function | Basis function optimized for non-linear driver fitting | Natural Cubic Spline (df = 3) |
|  | Lag-response Function | Basis function optimized for temporal distributed smoothing | Natural Cubic Spline (df = 3) |
|  | Maximum Lag Period | Boundary determined by eco-climatic seasonal cycles in Yunnan | 6 Months |

| **Table S2**. Robustness Check: Comparison between Baseline Poisson and NB Regressions | | | | |
| --- | --- | --- | --- | --- |
| Variable | Poisson RR | Poisson *p*-value | NB RR | NB *p*-value |
| LST | 0.86 | $<0.001$ | 0.88 | $<0.001$ |
| Precipitation | 0.84 | $<0.001$ | 0.85 | $<0.001$ |
| EVI | 0.749 | $<0.001$ | 0.75 | $<0.001$ |
| NDVI | 0.96 | 0.112 | 0.95 | 0.410 |
| NDWI | 1.94 | $<0.001$ | 1.887 | $<0.001$ |
| SoilMoist | 1.05 | $<0.001$ | 1.04 | $<0.001$ |
| NTL | 1.00 | 0.549 | 1.00 | 0.896 |
| Urban Area | 1.148 | $<0.001$ | 1.125 | $<0.001$ |
